# Using Serious Games for Antismoking Health Campaigns: Experimental Study

**DOI:** 10.2196/18528

**Published:** 2020-12-02

**Authors:** Jihyun Kim, Hayeon Song, Kelly Merrill Jr, Younbo Jung, Remi Junghuem Kwon

**Affiliations:** 1 University of Central Florida Orlando, FL United States; 2 Sungkyunkwan University Seoul Republic of Korea; 3 Ohio State University Columbus, OH United States; 4 Nanyang Technological University Singapore Singapore; 5 Korea University Technology and Education Cheoan-si Republic of Korea

**Keywords:** fear appeals, serious games, smoking, entertainment education, susceptibility

## Abstract

**Background:**

Serious games for health have been gaining in popularity among scholars and practitioners. However, there remain a few questions to be addressed.

**Objective:**

This study tests the effects of a serious game and fear appeals on smoking-related outcomes. More specifically, this research aims to understand how serious games function as a more effective vehicle for a health campaign than a traditional medium, such as a print-based pamphlet. Further, while serious games utilize a variety of persuasive strategies in the game’s content, it is not clear whether fear appeals, which are widely used persuasive-message strategies for health, can be an effective strategy in serious games. Thus, we are testing the effect of fear appeals in a serious game.

**Methods:**

We created a computer game and a print brochure to educate participants about the risks of smoking. More specifically, a flash-based single-player game was developed in which players were asked to avoid cigarettes in the gameplay context. We also developed an online brochure based on existing smoking-related brochures at a university health center; antismoking messages on the computer game and in the brochure were comparable. Then, an experiment using a 2 (media type: game vs. print) x 2 (fearful image: fear vs. no-fear) between-subjects design was conducted. The study recruitment was announced to undergraduate students enrolled in a large, public Midwestern university in the United States. After a screening test, a total of 72 smokers, who reported smoking in the past 30 days, participated in the experiment.

**Results:**

Overall, gameplay, when compared to print-based pamphlets, had greater impacts on attitudes toward smoking and the intention to quit smoking. Further, the game’s persuasive effects were especially pronounced when messages contained fear appeals. When fearful images were presented, participants in the game condition reported significantly more negative attitudes toward social smoking than those in the print condition [*F*(1,67)=7.28; *P*=.009; *η*_p^2^_=0.10]. However, in the no-fear condition, there was no significant difference between the conditions [*F*(1,67)=0.25; *P*=.620]. Similarly, the intention to quit smoking [*F*(1,67)=4.64; *P*=.035; *η*_p^2^_=0.07] and susceptibility [*F*(1,67)=6.92; *P*=.011; *η*_p^2^_=0.09] were also significantly different between the conditions, but only when fear appeals were used.

**Conclusions:**

This study extends fear appeal research by investigating the effects of different media types. It offers empirical evidence that a serious game can be an effective vehicle for fear appeals.

## Introduction

### Smoking and Smoking Cessation Interventions

Smoking is one of the most prevalent risky health behaviors. Currently, there are more than 35 million Americans that identify as smokers [1]. Although the number of smokers has consistently declined over the years, smoking has frequently been associated with negative health outcomes that often lead to death. Worldwide, smoking is responsible for more than 7 million deaths per year [2]. In the United States alone, more than 480,000 deaths a year are due to smoking, which includes 41,000 deaths due to second-hand smoking [3]. Researchers and medical professionals have also identified smoking as one of the most important risk factors for various health problems, such as different types of cancer [4], kidney failure [5], and cardiovascular disease [6]. In fact, more than 16 million Americans are living with a disease that was caused by smoking [3].

Smoking is particularly serious among young adults. Statistics indicate that nearly 9 out of 10 smokers try their first cigarette by the age of 18 years [3]. More than half of college smokers are reported as social smokers [7], and 1 in every 5 social smokers become a daily smoker during their 4 years at college [8]. In all, extant evidence suggests a strong need for investigating social smokers at the college level [9].

In an attempt to resolve prevalent smoking behaviors, much effort has focused on antismoking campaigns and interventions. For example, researchers have investigated the smoker identity to better understand how a smoker’s self-identification is related to their attitude toward smoking [9]. Also, the effectiveness of diverse media types for smoking cessation interventions have been examined, such as text messaging [10] and web-based approaches [11]. However, regardless of these efforts, smoking issues remain. This calls for a need to consider a different type of medium for health intervention, such as serious games for health.

Serious games for health have been gaining popularity among scholars and practitioners. However, there remain a few questions to be addressed. First, there is a strong need to understand how serious games can be more persuasive compared to other media types, such as print pamphlets. Second, while serious games utilize a variety of persuasive strategies in the game content, it is not clear whether fear appeals, which are widely used persuasive-message strategies for health, can be an effective strategy in serious games. That is, collectively, little research has examined the effects of media types when fear appeals have been utilized in the game content. To fill in the gap, this study investigates the effectiveness of a serious game compared to a printed brochure in the context of fear appeals.

### Fear Appeals

Fear appeals are described as “a persuasive message that attempts to arouse the emotion of fear by depicting a personally relevant and significant threat” [12]. That is, fear appeals are generally designed to scare people by describing negative outcomes that could occur when a recommended health behavior is not followed [12-14]. In acknowledgment of their effectiveness, fear appeals have been used as a core concept in a handful of health-related theories, such as the Protection Motivation Theory (PMT) [15] and the parallel process model [16]. Of the various fear-based theories, the extended parallel process model (EPPM) uses a more extensive approach to the understanding of fear [12] by combining aspects of PMT and the parallel process model. EPPM was developed to address the varying outcomes of these previous approaches regarding fear appeal messages. For example, research [12] emphasized “the role of the emotion fear in individuals’ responses to fear appeals” in EPPM, which was not fully addressed in other theories.

Fear appeals have specifically been used to combat health issues and produce positive outcomes; fear-based messages have been incorporated in diverse health campaign materials to persuade people to change their behaviors and attitudes toward making healthy choices [17]. Song et al [18] found that seeing a visualized fearful future, which was presented via a deteriorated image of one’s self due to smoking, increased not only smokers’ negative attitudes toward social smoking but also their intention to stop smoking when compared to other smokers not exposed to the fearful future. Findings from a meta-analysis also noted the effectiveness of fear appeals [14].

Although fear has been studied for decades, questions remain. In particular, it is not clear whether a fear-based strategy would also work effectively in gaming contexts. The effects of fear appeals were examined in various media platforms (eg, text, graphic/images, video); however, the role of fear in interactive games (eg, serious games) compared to other traditional media platforms (eg, print-based brochure) has not been fully addressed. In this regard, there is a need to understand how a fear-based strategy would work in games, compared to other traditional media platforms.

### Serious Games

Serious games are computer or video games designed for the primary purpose of educating users beyond entertainment purposes (eg, educational games, exercise video games, language learning games) [19-22]. Studies have demonstrated that serious games are effective in sharing information as well as changing attitudes and behaviors in a variety of health domains, such as safe sex [23], healthy eating [24-26], cancer [27,28], smoking [29,30], exercise [31-33], asthma management [34,35], and over-the-counter medication [36].

Several health education strategies are incorporated into serious games, including exposure therapy, behavioral rehearsal, and role-playing. The exposure therapy strategy provides repeated cues that trigger anxiety, often employed to diminish the anxiety or urge as a treatment for anxiety disorders such as acrophobia and arachnophobia [37-40]. The behavioral rehearsal strategy involves individuals practicing healthy behaviors, such as utilizing coping strategies to avoid relapse from smoking or choosing healthy foods repeatedly in a game to encourage the participant to engage in similar behaviors in a real-life setting [26]. Lastly, the role-playing strategy is used to help people understand various social settings from other perspectives [41].

### Features and Underlying Mechanisms of Serious Games

Games, compared to traditional media, have several unique built-in features. In particular, games can provide powerful learning experiences to change real-life behaviors with structural features and mechanisms that enable individuals to have their own experiences of the daunting future. In fact, research has identified various benefits of games for learning [42-47]. Specifically, Gee [43] highlighted certain features of games that are beneficial to learning. These include co-design and customization, where players have an interactive role with the content they engage with; manipulation, which allows players to engage in actions at a distance; and identity, where players have the ability to take on a new identity in games. These features allow the player to have new, meaningful experiences that have the potential to alter the player’s thought processes. The following describe some unique features and underlying mechanisms of serious games in more detail: (1) the combination of enactive and vicarious learning; (2) persuasion through entertainment education; and (3) interactively tailored content.

#### Combination of Enactive and Vicarious Learning

Social Cognitive Theory [48] explains that there are 2 types of human learning processes: enactive and vicarious learning. Enactive learning occurs when individuals directly experience the consequences of their behaviors. Vicarious learning occurs when individuals observe other people’s actions and thereby experience the consequences of their behaviors indirectly. Research [48,49] explains that enactive learning is more powerful than vicarious learning; however, enactive learning is often limited due to physical limitations (eg, time, space). Thus, vicarious learning is inevitable to expand humans’ experiences beyond everyday life routines. Someone else’s rare experiences or stories in the media (eg, movies, television) can provide a much wider range of occurrences that cannot be directly experienced.

Particularly, smoking-related fear appeals have been primarily associated with vicarious learning [50] because direct experiences of negative consequences could cause detrimental effects on the smoker’s real body. Thus, efforts have been focused on sharing someone else’s negative experiences of smoking via various methods such as images, messages, or testimonials. Research [49] argues that vicarious learning plays an important role by expanding knowledge and skills without necessarily having to participate directly in an action.

Following the above-mentioned argument, it is important to note that serious games can offer the benefits of both enactive and vicarious learning. First, the center of action resides in the game player rather than the observations of another individual’s actions. During gameplay, a player uses an individualized avatar (ie, game characters), and the player’s decisions and actions in the virtual environment have consequences for their avatar. In other words, players engage in enactive learning experiences through the body of the avatar. This process, conceptualized as “mediated enactive experience” [26], is a unique feature of gameplay compared to other traditional media where individuals passively observe someone else’s experiences. Additionally, Yee and Bailenson [51] found that people behave differently depending on the appearance of the avatar they control, which is understood as the Proteus Effect [51,52]. The Proteus Effect not only shows that the appearance of an avatar affects online behavior but also offline behavior [52]; an individual’s behavior in the real world can be altered based on the appearance of an avatar they control in a digital world. Thus, in-game actions that alter an avatar’s appearance not only influence the avatar but the individual controlling it as well.

Second, serious games also offer a breadth of vicarious learning. Unlike experiences in the physical environment, game experiences are not limited in terms of space, time, and the number of potential counterparts with whom players can interact. In gameplay, players can vicariously engage in actions and behaviors that may not be easily manifested in the physical world. Accordingly, a serious game is a unique vehicle; it provides a combination of both enactive and vicarious learning, can increase effective learning outcomes from the gameplay, and has the potential to be an effective persuasive medium.

#### Persuasion Through Entertainment Education

Fear appeals can be effective persuasive tools; however, they can also fail to accomplish what they were intended to do. One of the main reasons fear appeals can fail is that the experience of fear sometimes leads an individual to avoid or ignore a message that generates a negative emotion rather than changing risky behaviors [12]. Research has shown evidence that Entertainment-Education (E-E) can be successful by subverting topic avoidance caused by fear [53].

E-E is a strategy that utilizes popular entertainment media (eg, games, television, radio) to educate audiences [54,55]. The fun factor embedded in E-E materials generates intrinsic motivation and facilitates audience involvement that can result in persuasive message reception [56]. In a longitudinal qualitative study [57] focused on using a serious game to help with smoking cessation, participants indicated that they found the serious game to be fun; and these participants also demonstrated greater levels of motivation than the participants that were not exposed to a serious game.

For the same reason, people watch horror movies despite the negative emotions (eg, horror, sadness) they may experience, as the fun factor in the narratives holds an audience’s attention through the duration of the message [53,58]. For example, a suspense study [58] showed individuals were willing to experience negative emotions and intensive arousal as a payoff to know what would happen next in the narratives. Previous work also explains that narrative forms of communication may sustain attention among low-motivation audiences and generate inadvertent persuasive effects [59]. Further, E-E has been found to be persuasive by reducing counterarguments [53]. According to the Extended Elaboration Likelihood Model (E-ELM), viewing dramatic elements in contexts (eg, gory and fearful images) leads to less critical perceptions of content [60-62]. However, viewers are less likely to develop counterarguments as they encounter E-E messages and are therefore more likely to be persuaded by the messages.

#### Interactively Tailored Content

Messages can be more persuasive when they are tailored to the specific individual or group of people exposed to them. Tailoring is defined as “any combination of information or change strategies intended to reach one specific person based on characteristics unique to that person, related to the outcome of interest, and derived from an individual assessment” [63]. Thus, an advantage of tailored messages is their ability to depend on the individual differences among people, rather than assuming the effects are homogenous across a group of people [64].

Compared to nontailored messages, tailored messages are more likely to be read, understood, recalled, and perceived as trustworthy; thereby, they have a greater potential for persuasion and behavioral change [65-69]. Particularly, tailored messages have been effective in fear appeal strategies by motivating individuals to think that the risk condition may happen to them (ie, increasing perceived susceptibility) [70-72]. When tailored messages are provided, individuals tend to perceive that the messages are better fit for their condition [73,74], resulting in an increased level of perceived susceptibility. Important to note, however, is a meta-analysis using tailored health-behavior change interventions [75]. Results indicated that message tailoring has positive effects on most health-related attitudes except perceived susceptibility. While more research is needed to understand the results of the meta-analysis, a direction for future research would be testing the impact of tailored messages in varying degrees on perceived susceptibility.

As tailoring/customization is afforded in many digital and video games [76], serious games, too, can provide tailored health messages as well as personalized, interactive feedback associated with each action. For example, the game narratives develop based on a player’s decisions and behaviors in a highly interactive fashion. As Rafaeli [77] indicated, interactivity, considered to be a natural characteristic of face-to-face conversation (eg, communicating with a personal counselor), is now commonly observed in the computer-mediated environment. Games have considerable opportunities for message tailoring and providing interactive feedback at any time with almost no additional cost, unlike traditional, print-based formats that may have limitations both in quantity and quality of message tailoring.

In fact, empirical research has found that people exposed to a more interactive message were more likely to change their attitudes [30]. For instance, participants exposed to serious games are able to abstain from smoking longer than participants that were not exposed to serious games [78,79]. This is likely due to gamified application, which helps to distract participants from smoking and provided helpful smoking cessation advice [80]. Raiff et al [81] also found that participants are more likely to use a serious game to aid the cessation of smoking as compared to other cessation tactics, such as using a nicotine patch, a drug, or attending a support group.

This study compares the potential of a serious game to a traditional, print-based format regarding the use of fear appeals as a persuasive vehicle. More specifically, the current study hypothesizes that antismoking messages featured in a serious game will be more likely to positively influence smoking-related outcomes than a print-based brochure. Additionally, the study tests the role of fear appeals in serious games to understand whether media types would interplay with fear appeal strategies. To better understand the key aspects of smoking-related outcomes, the study specifically focuses on the following: attitudes toward smoking, the intention to quit smoking, and perceived susceptibility.

Based on the aforementioned literature, the following 3-part hypothesis is proposed: Individuals playing an antismoking game report stronger levels of (1) negative attitudes toward social smoking, (2) intention to quit smoking, and (3) perceived susceptibility to smoking-induced risks than individuals reading an antismoking print-based pamphlet. The following research question is also proposed: What role does a fear appeal strategy play in a serious game compared to a print brochure for an antismoking health campaign?

## Methods

### Participants

To identify eligible participants, a screening test was conducted online among undergraduate students enrolled in a large, public Midwestern university in the United States. Only individuals who reported smoking in the past 30 days were contacted. Thus, a total of 72 smokers were included in this study. Of the 72 participants, the sample comprised of 41 men (57%) and 31 women (43%), with a mean age of 21.40 (SD 5.14) years. The median smoking frequency was 2 to 3 times a week.

We conducted an experiment to test the hypothesis and research question. In particular, a 2 (media type: game vs. print) x 2 (fearful image: present vs. not present) between-subjects design was employed. Participants were assigned into 1 of the 4 experimental conditions: the game-fear condition (n=16), the game–no fear condition (n=16), the print-fear condition (n=19), and the print-no-fear condition (n=21). To ensure that there were no initial differences among the experimental groups, several analyses were conducted regarding sex, age, and smoking frequency. Results found that there were no statistically significant differences between groups. Thus, group equivalence was ensured. Demographic information for each experimental group is presented in Table 1.

**Table 1:** Description of the participants (n=72) in each experimental group.

Participant characteristics		Game-fear group (n=16)	Game–no fear group (n=16)	Print-fear group (n=19)	Print–no fear group (n=21)
**Gender^a^, n**					
	Male	11	9	9	12
	Female	5	7	10	9
Age^b^ in years, mean (SD)		19.81 (6.49)	21.88 (2.31)	22.58 (4.49)	21.19 (6.05)
Smoking frequency^c^, median times per week		1	2-3	2-3	2-3

^a^Group difference for sex: χ^2^(3)=1.624; *P*=.654.

^b^Group difference for age: *F*(3)=0.894; *P*=.449.

^c^Group difference for smoking frequency: χ^2^(18)=15.014; *P*=.661.

### Material Development

**Figure 1:**
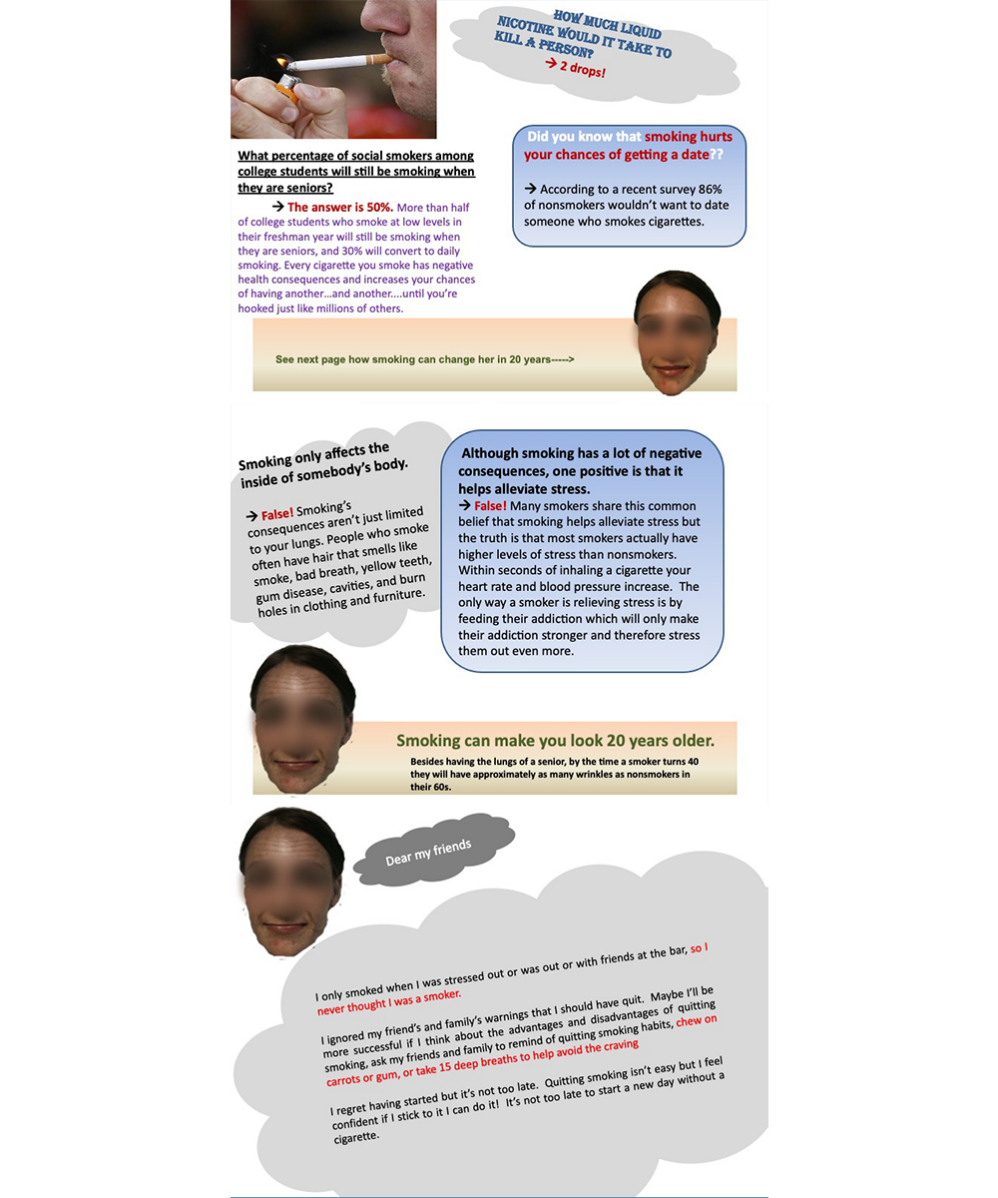
Antismoking intervention brochure. (The face of the avatar was not blurred in the original brochure; it has been blurred for this publication only).

A series of steps were taken to develop materials for the experiment. First, a print brochure was created (Figure 1). To develop health messages for the brochure, all the antismoking related brochures available at the health center on the university campus were collected. Factoids, or information frequently appearing in the brochures, were identified and reformatted into a question-and-answer format containing 8 questions and answers. Then, a smoker’s testimonial message, including feelings of physical weakness, regrets about not quitting smoking earlier, and complaints about looking old compared to nonsmokers of similar age, was created. The testimonial content was presented alongside a photo of the smoker in the brochure.

Fear conditions were manipulated based on the smoker’s face. In the no-fear condition, only a young and healthy-looking face of a smoker was presented. However, in the fear condition, both a young, healthy-looking face of a smoker and an old, wrinkled face of the same smoker (which was caused by smoking) were presented.

In the fear condition for the print brochure, one page of the pamphlet showed a young and healthy-looking face with the subtitle, “Go to the next page to see how smoking can change her.” On the following page, a discolored and wrinkled face was featured with a message stating, “Smoking can make you look 20 years older.” Further explanation was provided: “Besides having the lungs of a senior, by the time a smoker turns 40, they will have approximately as many wrinkles as nonsmokers in their 60s.” In the no-fear condition, only a young and healthy-looking face of a smoker was presented, and there was no mention of what smoking can change in a person’s face. Otherwise, all the textual and graphical messages remained the same as the fear condition.

**Figure 2:**
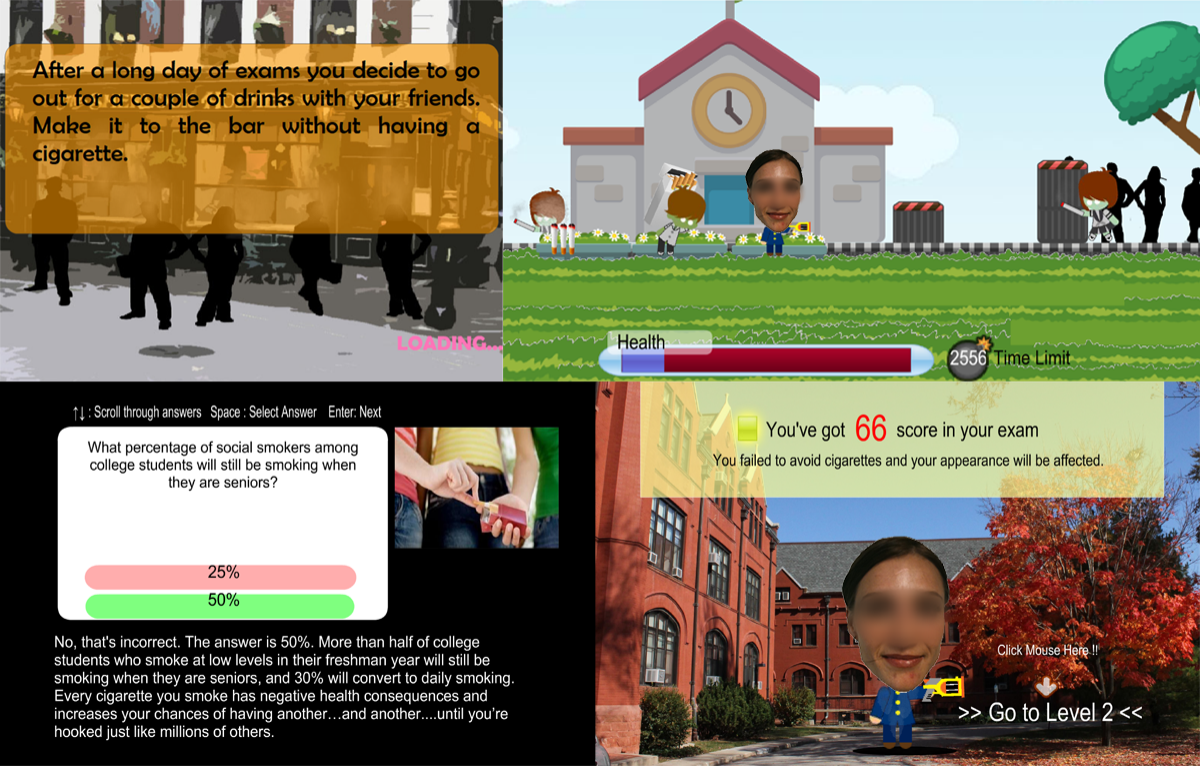
Antismoking intervention computer game. (The face of the avatar was not blurred in the original game; it has been blurred for this publication only).

For the game condition (Figure 2), a computer game was developed for the purpose of educating smoking risks. The game is a flash-based single-player game where users are asked to avoid smoking cigarettes when they are stressed out because of an upcoming exam (Level 1) and when they are hanging out at a bar (Level 2). This game includes all 3 structural features of digital games that were discussed earlier (ie, combination of enactive and vicarious learning, entertainment education, and interactive tailoring). Players have full control of the avatar, enabling mediated enactive experiences. All of the actions (eg, avoiding smoking) are based on the player’s own decisions. Players are in the center of the narratives and experience the negative consequences of the smokers (eg, not getting a second date, physical weakness, financial consequences) as their own experience rather than as someone else’s (ie, combination of mediated enactive and vicarious experience). The game ends with a monograph where the avatar regrets not quitting smoking while he or she was young. At the end of each level of the game, there is an educational quiz session, which was designed to be interactive. Depending on which answer the individual picks, true/false results are given, followed by a tailored explanation to give more detailed information about smoking. The game is designed to be easy to play—all of the participants in the game condition self-reported playing the game without difficulty.

In the fear condition for the game, participants played with an avatar that had a young and healthy-looking face in Level 1. Then, they were told their avatar’s face would look aged in Level 2 due to smoking experienced in Level 1. In the no-fear condition, a young and healthy-looking face was used in both Levels 1 and 2. In order to avoid any potential confounding effects, the gender of the smoker’s face was matched with that of participants in both conditions. Also, the smoking-related content portrayed in the game was consistent with the print pamphlet.

### Procedure

Upon the university’s Institutional Review Board’s approval, a recruitment message was distributed via the university’s email system. Interested participants contacted the researcher via an online survey system. Prior to the experiment, researchers conducted a pretest to assess baseline smoking-related attitudes and perceptions. Approximately 2 weeks after the completion of the pretest, half of the participants, who were randomly selected to a game condition, were invited to a physical lab where they played the game. The other half of the participants were invited to an online lab where they viewed the pamphlet. Upon completing the main task of the study, participants were instructed to complete a posttest.

### Measures

#### Pretest

Negative attitudes toward social smoking (*α*=.77) were measured by 6 items (eg, “Social smoking can cause health issues”). Intention to quit smoking (*α*=.89) was assessed using 8 items (eg, “I am willing to try quitting smoking”). All of the responses were obtained on a 10-point Likert-type scale (1=*Strongly Disagree*; 10=*Strongly Agree*).

#### Posttest

Negative attitudes toward social smoking (*α*=.77) and intention to quit smoking (*α*=.89) were measured in the posttest again, and the same items were used from the pretest. Susceptibility (*α*=.89) was measured by 6 items focusing on the negative consequences of smoking described in the antismoking messages (eg, “I think I will look old if I keep smoking” and “I know that I will have bad breath if I keep smoking”). Due to the specificity of the measure related to the messages in both the game and print conditions, considerable test-sensitization issues were expected. Therefore, susceptibility was asked only once in the posttest. All of the measures were developed for the study, and the responses were obtained on a 10-point Likert-type scale (1=*Strongly Disagree*; 10=*Strongly Agree*).

## Results

Controlling for the sex of the participants, a series of analyses of covariance (ANCOVAs) were conducted using SPSS software (version 25; IBM Corp) to test the proposed hypothesis and the research question. Because attitudes towards social smoking and intention to quit smoking were measured at both pretests and posttests, they were tested based on the score difference between pretests and posttests (score=posttest – pretest). Therefore, positive scores indicate that posttest scores are higher than the pretest scores. Since susceptibility was measured in the posttest only, the final index was based on the posttest score.

Regarding the first part of the hypothesis (hypothesis 1), individuals in the game condition (mean 0.93, SD 1.60), compared to those in the print condition (mean 0.09, SD 1.45), reported significantly more negative attitudes toward social smoking [*F*(1,67)=5.25; *P*=.03; *η*_p^2^_=0.07]. As for the second part of the hypothesis (hypothesis 2), there was no significant difference in the intention to quit smoking between the game condition (mean 0.49, SD 1.17) and the print condition (mean -0.22, SD 1.99) [*F*(1,67)=2.83; *P*=.10; *η*_p^2^_=0.04]. With regard to the third part of the hypothesis (hypothesis 3), individuals in the game condition (mean 8.48; SD 1.22) reported a greater level of susceptibility [*F*(1,67)=5.81; *P*=.02; *η*_p^2^_=.08] than those in the print condition (mean 7.65, SD 1.91). In sum, hypothesis 1 (attitudes) and hypothesis 3 (susceptibility) were supported, but hypothesis 2 (intention) was not. Table 2 illustrates the main effects of media format and fear.

**Table 2:** Main effects of media format and fear.

Condition		Condition, mean (SD)				Univariate *F*	*P* value	*η*_p^2^_
		Game	Print	Fear	No fear			
**Media format**								
	Attitudes	0.93 (1.60)	0.09 (1.45)	N/A^a^	N/A	5.25	.03	.07
	Intention	0.49 (1.17)	-0.22 (1.99)	N/A	N/A	2.83	.10	.04
	Susceptibility	8.48 (1.22)	7.65 (1.91)	N/A	N/A	5.81	.02	.08
**Fear**								
	Attitudes	N/A	N/A	0.60 (1.38)	0.33 (1.72)	0.78	.38	.01
	Intention	N/A	N/A	0.15 (1.91)	0.05 (1.50)	0.13	.72	.002
	Susceptibility	N/A	N/A	7.61 (2.01)	8.41 (1.21)	4.16	.045	.06

^a^N/A: not applicable.

To answer the research question that examined the interplay between media types and fear appeals, interaction effects and simple main effects were assessed (Table 3).

None of the interaction effects were significant [attitudes: *F*(1,67)=2.52, *P*=.12; intention: *F*(1,67)=2.00, *P*=.16; susceptibility: *F*(1,67)=1.89, *P*=.17]. However, the differences between the pretests and posttests appeared to be greater in the game condition compared to the print condition (Table 3).

Further analyses were conducted to examine the simple main effects of fear and media types. As 2 different comparisons were conducted in each simple test, the decision of statistical significance was made based on the adjusted *P* value .025 (.05/2) to reduce any potential error rate. First, when fearful images were presented, participants in the game condition reported significantly more negative attitudes toward social smoking than those in the print condition [*F*(1,67)=7.28; *P=*.009; *η*_p^2^_=0.10]. However, in the no-fear condition, there was no significant difference between the game and the print condition *[F*(1,67)=0.25; *P=*.62]. Further, there was no significant difference between the fear and no-fear condition in the game condition [*F*(1,67)=2.76; *P=*.10] and in the print condition [*F*(1,67)=0.29; *P=*.60]. In all, the result indicated that the effect of fear on negative attitudes toward social smoking was stronger in the game condition than the print condition.

Next, simple main effects on the intention to quit smoking were tested. In the fear condition, participants in the game condition reported greater intention at a *P* value of .035 [*F*(1,67)=4.64; *P*=.035; *η*_p^2^_=0.07] compared to those in the print condition. However, in the no-fear condition, there was no significant difference [*F*(1,67)=0.04; *P=*.85]. Further, there were no significant differences between the fear and no-fear condition in the game condition [*F*(1,67)=1.42; *P=*.24] and in the print condition [*F*(1,67)=0.63; *P=*.43]. The result found that the effect of fear on the intention to quit smoking was stronger in the game condition than the print condition, although the *P* value was at .035.

**Table 3:** Simple effects of media format and fear.

Condition		Condition, mean (SD)				Univariate *F*	*P* value	η_p^2^_
		Game	Print	Fear	No fear			
**Fear**								
	Attitudes	1.40 (1.02)	-0.06 (1.32)	N/A^a^	N/A	7.28	.009	.10
	Intention	0.87 (1.23)	-0.46 (2.19)	N/A	N/A	4.64	.035	.07
	Susceptibility	8.33 (1.52)	7.00 (2.20)	N/A	N/A	6.93	.011	.09
**No fear**								
	Attitudes	0.47 (1.94)	0.22 (1.58)	N/A	N/A	0.25	.62	.004
	Intention	0.11 (1.50)	0.01 (1.81)	N/A	N/A	0.04	.85	.001
	Susceptibility	8.64 (0.84)	8.24 (1.42)	N/A	N/A	0.56	.46	.01
**Game**								
	Attitudes	N/A	N/A	1.39 (1.02)	0.47 (1.94)	2.76	.10	.04
	Intention	N/A	N/A	0.87 (1.23)	0.11 (1.01)	1.42	.24	.02
	Susceptibility	N/A	N/A	8.33 (1.52)	8.64 (0.84)	0.19	.66	.003
**Print**								
	Attitudes	N/A	N/A	-0.06 (1.32)	0.22 (1.58)	0.29	.60	.004
	Intention	N/A	N/A	-0.46 (2.19)	0.01 (1.81)	0.63	.43	.01
	Susceptibility	N/A	N/A	7.00 (2.20)	8.24 (1.42)	6.54	.013	.09

^a^N/A: not applicable.

Regarding susceptibility, when fear was presented, participants in the game condition reported greater susceptibility than those in the print condition [*F*(1,67)=6.92; *P=*.011; *η*_p^2^_= 0.09]. However, when fear was not presented, there was no significant difference between the 2 media types [*F*(1,67)=0.56; *P=*.46]. Further, in the game condition, there was no significant difference between the fear and no-fear condition [*F*(1,67)=0.19; *P=*.66]. Finally, in the print condition, people in the no-fear condition reported greater susceptibility than those in the fear condition [*F*(1,67)=6.54; *P=*.013; *η*_p^2^_=0.09]. The results indicated that the effect of fear on susceptibility was stronger in the game condition than the print condition. Further, in the print condition, not using fear was more effective than using fear in increasing susceptibility (Table 3).

## Discussion

### Principal Findings

This study compared the effectiveness of game- and print-based fear appeal messages in the context of social smoking, such as attitudes toward smoking, intention for smoking cessation, and susceptibility. Overall, the results indicate that games can be a more persuasive vehicle than print-based pamphlets when fear is incorporated in the message. Specifically, the study found that when smokers play the game, they experience more negative attitudes toward social smoking and greater susceptibility than those who read the pamphlet. Further, the result of the simple main effect analyses suggest that the game’s persuasive effects are more pronounced when the media content includes fear messages; when a fearful image is presented in the form of an altered, aged face due to smoking, playing the game is significantly more likely to induce persuasive outcomes compared to reading the print pamphlet. However, such differences between the 2 media types are not observed when the fearful image is not presented.

### Contributions and Implications

The present study extends fear appeal research by investigating the persuasive role of games and offers empirical evidence that a serious game can be an effective vehicle for fear appeal strategies. Games can provide unique opportunities that other traditional media cannot offer, and the use of games has the potential to further expand the fear appeal literature. For example, games can be an effective medium to identify and design an optimal situation to make fear appeal strategies work effectively, as game features make it possible to manipulate the characteristics of the avatar or narratives in order to enhance the effects of fear appeals. In addition, games can be tailored to each participant at reasonable costs. Serious games implemented in settings such as waiting rooms at hospitals and health care centers, for instance, may provide more successful outcomes than placing traditional pamphlets on magazine racks.

Another contribution of this study is the findings related to the effect of media types on susceptibility. Previous literature shows that promoting susceptibility has often been challenging because susceptibility is vulnerable to the innate human tendency to possess an optimistic bias [82]. Individuals are likely to prospect that their own future will be brighter compared to average people, which can interfere with a person’s accurate assessment of health risks [83]. In a similar vein, a meta-analysis [75] revealed that tailored messages, which are widely considered to be an effective strategy in health campaigns, resulted in positive changes for all other health-related attitudes except susceptibility. The meta-analysis study found that susceptibility demonstrated the opposite pattern; tailored messages reduced perceptions of susceptibility. A plausible explanation is that the attempt to tailor messages in traditional formats of media is limited in its ability to trigger enactive learning experiences, which sometimes results in the boomerang effect of reduced susceptibility. However, the findings of our study imply that serious games may enhance the perception of susceptibility more than print-based brochures. In fact, this finding is supported by research that interactive media enhances perceived susceptibility [71]. As such, this study argues that the interplay among susceptibility, media types, and fear appeals is important to note and calls for more research.

In addition, this study further strengthens the extant body of research. While a handful of studies have already investigated serious games and smoking, the current investigation is unique from the others in various ways. Specifically, this study examines additional key variables, as compared to the previous studies. In particular, one of the foci of this investigation is on susceptibility, which has received relatively little attention in previous game studies, except in the Song et al [18] study. Also, the present investigation has more potential to generalize the findings to a broader population. While most of the studies similarly indicated that participants had to be at least 18 years old and active smokers, some focused on specific populations, such as pregnant women [79], previously diagnosed cancer patients [78], and individuals identified as having a mental illness [84]. Some studies employed even more requirements. For example, participants had to either own a smartphone [57,79], score above a specific criterion for motivation to stop smoking [79], or had to be currently taking psychiatric medicine, as well as have an assigned psychiatric case manager and psychiatric provider [84]. While these studies provide specific information for particular groups of smokers, the implications of the study interventions might be somewhat limited to a targeted group. In this regard, this study has the potential to generalize results more broadly, as participants were not limited to particular conditions. Moreover, this study effectively compares differences in key variables between a printed pamphlet and a serious game. While some studies (such as the one conducted by Vilardaga et al [84]) tested a paper prototype of their serious game to aid in the development of their actual serious game, the comparison of a serious game and some form of a paper pamphlet is largely missing from previous studies.

Furthermore, this study offers implications for practice. Although an increasing number of studies have reported the effectiveness of games, relatively few have compared the effects of serious games for health education compared to the effects of traditional, printed brochures in the context of fear messages. In this regard, this study demonstrates empirical evidence that health practitioners, especially those who are planning to use fear appeal strategies, should pay close attention to interactive media such as serious games. The study’s findings imply that the threat induced by a comparable description of the dire consequences of current health behaviors can be used more effectively to change problematic behaviors when a game, compared to printed brochures, is utilized. Games can provide powerful experiences to change real-life behaviors with their structural features [85], which can also enable individuals to have their own experience of the daunting future that has not yet materialized in a highly tailored and fun way, paradoxically.

Additionally, this study suggests the adoption of serious games, especially when the intervention is targeting individuals with a low-risk perception. For example, social smokers do not tend to believe that they will suffer from the negative consequences of smoking and even fail to identify themselves as smokers [86]. Thus, interventions targeting regular or habitual smokers are easily neglected by social or occasional smokers. In this regard, games tend to be more approachable to them, and they can be more persuasive.

However, it is important to note that this study does not always suggest using serious games. As part of this study’s findings indicate, there was no significant difference between the 2 media types in the no-fear condition. This result implies that different media types are likely to provide maximum effectiveness when their unique strengths are considered in developing campaigns. For example, print brochures may be useful for providing simple information about health behaviors, while serious games may be helpful for increasing perceived susceptibility in fear appeal messages. It is also important to acknowledge cost-related issues. To avoid offsetting monetary challenges, one could use print brochures to provide simple information about health behaviors for a relatively cheap cost. However, serious games, compared to printed brochures, may be more helpful for increasing perceived susceptibility in fear appeal messages. Then, a free app on a smartphone could make serious games more accessible to the general public. Future research needs to explore various ways to utilize diverse media types in order to maximize the effectiveness of health campaigns.

### Limitations and Future Research Directions

As with any research, this study also has a few limitations that should be considered when interpreting the results. First, the study utilized fearful images to investigate the effect of fear appeals but did not consider other types of fear appeal strategies. Given that fear appeals are multifaceted stimuli [15], research has indicated a variety of factors that may influence the effects of fear appeal strategies such as narratives. In order to further expand the study’s findings, future research should investigate various stimuli or the combination of multiple stimuli for fear appeals. In that way, the findings may help design more effective fear appeal strategies by providing detailed practical information.

Second, the study acknowledges that different ranges of exposure time to the study’s stimulus may have influenced the results. Although the message presented in each medium (game and print) was comparable, it was not possible to control the time that each participant spent in their experimental condition. In particular, due to the nature of the medium, participants in the game condition may have spent a bit more time completing their tasks than those in the print condition. Even in the game condition, some participants may have finished the gameplay faster than others. In this regard, future researchers are encouraged to further investigate this issue.

Next, participants in this study were all college students. While previous research has already identified social smoking as being a prevalent behavior among college students [7-9], many others are engaging in this risky health behavior [78,79,84]. Thus, it is possible that the results of the study might be limited to this college student population. Future research should incorporate a more representative sample to ensure these results are more generalizable.

Moreover, the study focused on short-term effects only. Participants were asked their attitudes toward smoking only after interacting with either the game or the pamphlet. Thus, it is not clear whether these interactions would have a long-term effect on the participant’s behavior. Future research should conduct a longitudinal study to assess how effective games and pamphlets are at inducing behavioral changes, such as intentions to quit smoking, over time.

Lastly, although this study did not investigate the role of narratives specifically, the study acknowledges that narratives in games play an important role, especially in persuasion [87]. With the coined term “procedural rhetoric,” Bogost [87] explains that the users of persuasive games learn through the authorship of rules and processes by becoming the game character. Bogost [87] argues that learning achieved by experiencing the procedural rhetoric in persuasive games is quite different from learning-based simply on words/texts and visual images. Similarly, the concept of narrative persuasion has also been conceptualized and tested together with the feeling of transportation in persuasion and education contexts [88,89]. While these concepts may have played a role in this study’s findings, they were not empirically tested in this investigation. Future researchers are encouraged to further expand this line of research.

### Conclusion

Games have great potential to expand fear appeal research with their unique features. As with media convergence, games are gradually merged into other media such as social media and social television. Thus, it is important to further investigate ways to utilize games merged into other media to expand the fear appeal literature. In this regard, this study sheds light on the use of serious games in the context of utilizing fear appeals.

## Abbreviations

E-E: Entertainment-Education
EPPM: extended parallel process model
PMT: Protection Motivation Theory
