# Correction: Using Serious Games for Antismoking Health Campaigns: Experimental Study

**DOI:** 10.2196/28180

**Published:** 2021-03-08

**Authors:** Jihyun Kim, Hayeon Song, Kelly Merrill Jr, Younbo Jung, Remi Junghuem Kwon

**Affiliations:** 1 University of Central Florida Orlando, FL United States; 2 Sungkyunkwan University Seoul Republic of Korea; 3 Ohio State University Columbus, OH United States; 4 Nanyang Technological University Singapore Singapore; 5 Korea University Technology and Education Cheoan-si Republic of Korea

In “Using Serious Games for Antismoking Health Campaigns: Experimental Study” (JMIR Serious Games 2020, 8(4):e18528) the authors noted one error.

The Acknowledgments section of the original article was published as follows:

This research was supported by the MSIT (Ministry of Science and ICT), Korea, under the ICAN (ICT Challenge and Advanced Network of HRD) program (IITP-2020-0-01816) supervised by the IITP (Institute of Information & Communications Technology Planning & Evaluation).

An additional funding source has been added to the Acknowledgments section, which now reads:

This research was supported by the MSIT (Ministry of Science and ICT), Korea, under the ICAN (ICT Challenge and Advanced Network of HRD) program (IITP-2020-0-01816) supervised by the IITP (Institute of Information & Communications Technology Planning & Evaluation).

This work was supported by the Ministry of Education of the Republic of Korea and the National Research Foundation of Korea (NRF-2019S1A5A2A03035424).

The correction will appear in the online version of the paper on the JMIR Publications website on March 8, 2021, together with the publication of this correction notice. Because this was made after submission to PubMed, PubMed Central, and other full-text repositories, the corrected article has also been resubmitted to those repositories.

